# Activity Levels in Healthy Older Adults: Implications for Joint Arthroplasty

**DOI:** 10.5402/2012/727950

**Published:** 2012-09-12

**Authors:** Laura E. Thorp, Diego Orozco, Joel A. Block, Dale R. Sumner, Markus A. Wimmer

**Affiliations:** ^1^Department of Anatomy and Cell Biology, Rush University Medical Center, 600 South Paulina Street, Armour Academic Facilities, Chicago, IL 60612, USA; ^2^Section of Rheumatology, Rush University Medical Center, 600 South Paulina Street, Armour Academic Facilities, Chicago, IL 60612, USA; ^3^Department of Orthopaedic Surgery, Rush University Medical Center, 600 South Paulina Street, Armour Academic Facilities, Chicago, IL 60612, USA

## Abstract

This work evaluated activity levels in a group of healthy older adults to establish a target activity level for adults of similar age after total joint arthroplasty (TJA). With the decreasing age of TJA patients, it is essential to have a reference for activity level in younger patients as activity level affects quality of life and implant design. 54 asymptomatic, healthy older adults with no clinical evidence of lower extremity OA participated. The main outcome measure, average daily step count, was measured using an accelerometer-based activity monitor. On average the group took 8813 ± 3611 steps per day, approximately 4000 more steps per day than has been previously reported in patients following total joint arthroplasty. The present work provides a reference for activity after joint arthroplasty which is relevant given the projected number of people under the age of 65 who will undergo joint arthroplasty in the coming years.

## 1. Introduction

It is well accepted that levels of physical activity in older adults relate to physical and cognitive performance measures. Physical activity is promoted as beneficial for a variety of diseases affecting middle-aged and older adults. Activity levels, specifically locomotor activities, are also relevant to total joint arthroplasty (TJA) surgery, both for the design of implants and for rehabilitation programs where postsurgical activity level is considered to be an important clinical outcome measure. In addition, differences in activity levels have been shown to affect the rate of polyethylene wear [1], a critical factor in implant longevity. Specifically, walking cycles have been reported to influence wear rates of polyethylene implants [2, 3]. 

Activity levels in patients after total hip and knee arthroplasty have been studied extensively, and have been reported to average approximately 5,000 steps per day in these patients [4–6], with one report suggesting that younger patients may attain an average of 6000 steps per day [7]. In contrast, the currently recommended goal for daily physical activity in adults is 10,000 steps per day, or 30 minutes of brisk walking [8]. A meta-analysis examining step counts assessed by pedometer in adults found that activity levels are related to age with activity in adults 18–65 approaching the 10,000-step recommendation while in those over 65 the mean daily step count was approximately 6500 steps [9]. While much can be gained from this meta-analysis it does not provide information specifically concerning activity levels in healthy older adults who are free from clinical evidence of osteoarthritis in the lower extremities. Such information would provide a realistic benchmark for activity levels in patients after joint replacement who often have expectations of returning to similar activity levels as their peers. 

This study sought to expand on existing literature regarding activity levels in adults by (1) characterizing the activity level of an older adult population without evidence of clinical osteoarthritis in the lower extremities, and (2) examining what factors might explain variance in activity levels in this population. We specifically targeted healthy adults between the ages of 45 and 70 as future projections indicate that this age group will comprise a majority of total joint arthroplasty patients in the coming years [10]. In addition, we utilized an accelerometer-based activity monitor rather than pedometers which have been reported to vary in their accuracy [11] and monitored the subjects over a seven-day period so as to capture a representative daily step count that is derived from activity on both weekdays and weekends. 

## 2. Methods

Healthy, asymptomatic subjects aged 45 to 70 years, and with no history of fracture or surgery involving the lower extremities, were recruited for participation in the present study through advertisement and word of mouth with the approval of the Institutional Review Board at Rush University. All subjects gave informed consent prior to participation. Subjects at any level of physical activity were encouraged to participate. Inclusion criteria were as follows: no clinical evidence of osteoarthritis in the lower extremities, no pain in the lower extremities, and no past history of surgery involving the hip, knee, or ankle bilaterally. To objectively assess pain in the hips and knees, subjects completed question no. 1 of the Western Ontario and MacMaster Universities Osteoarthritis Index (WOMAC) [12] which asks subjects to rate their pain in hip and knee joints on a visual analog scale (VAS). Subjects were included and classified as asymptomatic if they reported less than 10 mm (of a maximum of 100 mm) of lower extremity pain bilaterally at the hip and knee joints during level walking in response to this question. 41 women and 13 men (out of 65 total recruited) met the inclusion criteria for the study and were compliant with wearing the activity monitoring device for a seven-day period. The subject's strength and range of motion were assessed through gross manual muscle testing procedures and by goniometry by a trained physical therapist. All subjects exhibited strength and range of motion in the lower extremities that were within normal limits. 

A standard gait analysis protocol was employed to obtain data on walking speed for each subject in the laboratory setting. All tests were conducted by the same, trained individual. Briefly, four optoelectronic cameras (Qualysis, Gotenborg, Sweden) tracked the motion of six passive retroreflective markers, placed at the iliac crest, greater trochanter, lateral knee joint line, lateral malleolus, lateral aspect of calcaneus, and the head of the fifth metatarsal. Sagittal plane kinematics were calculated from the marker positions. Seven trials were collected for each subject, three trials at a self-selected “normal speed”, and two each at self-selected “fast” and “slow” speeds. Data presented in the present work correspond to the “self-selected” normal speed for each subject, so as to replicate normally walking as closely as possible in the laboratory setting. 

Subjects donned an accelerometer-based activity monitoring device (the AMP 331 Dynastream, Innovations, Cochrane, AB) on their right ankle. Each subject wore the activity monitoring device for a period of seven consecutive days during all waking hours except when showering or bathing. The device calculates the number of steps taken during level walking excluding stair-climbing activities. The total number of steps taken during the one-week period (7 consecutive days) was obtained, and a mean daily step count was computed. The one-week time period was chosen to ensure that week days and weekend days were both included for all subjects. Data were collected between May and September. 

Step count accuracy of the activity monitor was validated against an optical tracking system (Qualisys, Innovision Systems, Inc., Columbiaville, MI). In order to evaluate walking under normal conditions, eleven (5F/6 M) healthy, non-TJA volunteers age 28.8 ± 5.5 (range: 19–41) years old and a BMI of 24.9 ± 5.4 kg/m^2^ (range: 19.9–39.0 kg/m^2^) were asked to wear the activity monitor while walking on a treadmill (Motion Analysis Corp, California) at a normal self-selected speed (1.1 ± 0.2 m/s, 0.7–1.4 m/s). In addition to the activity monitor, two reflective markers were placed on the toes and heel area of the left and right feet, allowing the tracking and determination of steps. Step count for both systems was synchronized. Each volunteer was asked to walk a fixed distance of 300 meters. Acceleration, top self-selected speed, deceleration, and travel distance of the treadmill were adjusted and programmed for each volunteer. 

Intraclass correlation coefficients (ICC (2,1)) were used to determine whether data from the activity monitor were in agreement with the optical tracking system. An ICC greater than 0.7 indicated the two measurements were statistically similar. In order to graphically depict the goodness of agreement, Bland-Altman plots were generated, in which the average difference (=“bias”) of the two methods is plotted against the mean of the two measurements. The limits of agreement were computed as bias ±1.96 standard deviation of the difference. 

**Figure 1:**
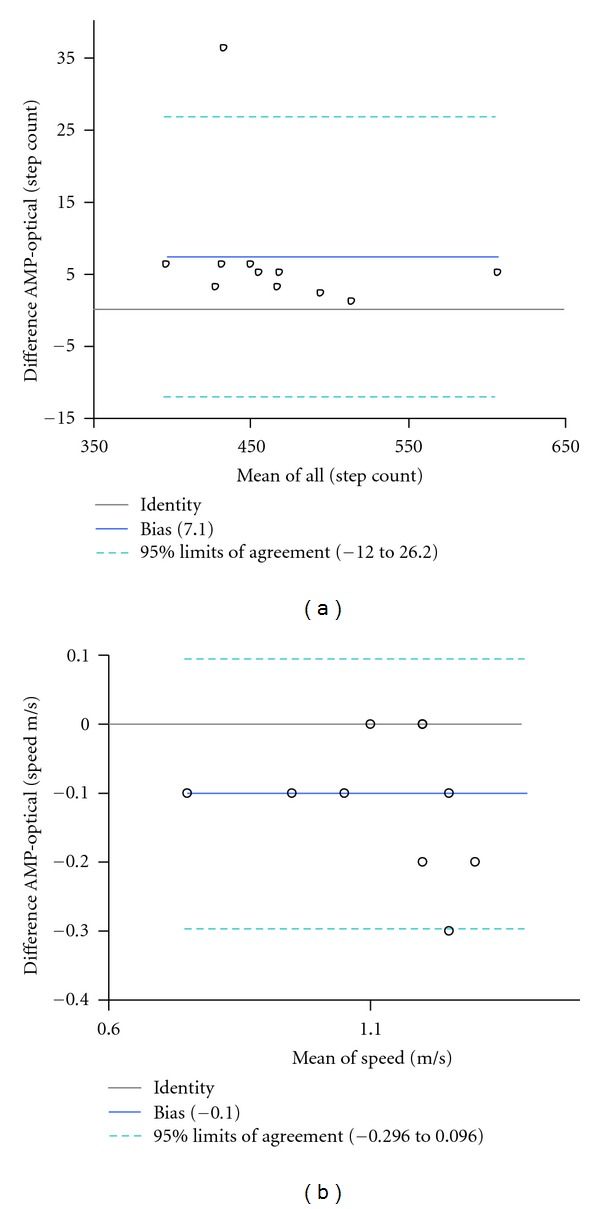
Bland-Altman plots depicting the error for (a) step count, and (b) speed between AMP activity monitor and optical tracking system.

 The ICC (2,1) between activity monitor and optical tracking system was 0.98 for step count and 0.76 for speed. This suggested that the activity monitor was accurate enough to count steps under normal walking conditions for a variety of body mass indices. Also speed was reasonably well correlated with the optical tracking system. As shown in Figure 1(a), the step monitor slightly overestimated the actual number of steps. However, the bias was typically smaller than 1.5%. In contrast, the speed was underestimated by the monitor in most cases, on average by 9% (Figure 1(b)).

A repeated measures ANOVA was used to examine day-to-day variability in step counts for the group as a whole. Pearson correlations were utilized to examine the relationship between age, BMI, gait speed, and average daily step count for the study population as a whole. In addition, a backwards elimination regression analysis was conducted with average daily step count as the dependent variable and age, gender, and BMI as independent variables to assess how these factors might simultaneously explain variance in activity level. Subjects were also subdivided into three groups based on their mean number of steps per day according to the classification scheme outlined by Tudor-Locke and Bassett [13]. Subjects who averaged less than 7500 steps per day were considered as a “sedentary—low activity group,” those who averaged between 7500 and 9999 steps per day were considered a “somewhat active group,” and those who averaged greater than 10,000 steps per day were considered to be an “active—highly active group” [13]. Between-group comparisons of demographic data and gait parameters were made using an analysis of variance with Bonferroni *post hoc* tests. All statistical calculations were carried out using SPSS statistical software version 13.0 (Chicago, IL).

## 3. Results 

**Figure 2:**
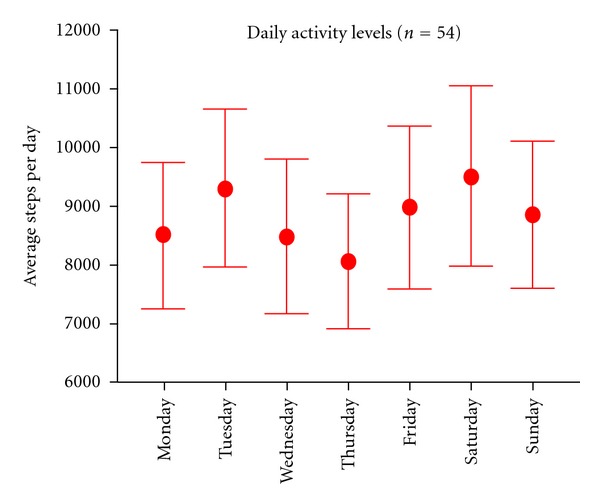
Mean daily step count for the group as a whole plotted as a function of day of the week. Circles represent the mean step count for all 54 subjects on a given week day and bars represent standard deviations.

Activity data were collected on fifty-four subjects (41 women and 13 men) who met inclusion criteria for participation and were compliant wearing the monitor for seven days. The mean (±SD) age of the subjects was 54.1 ± 6.1 years, with a range of 45 to 68 years. The mean (±SD) body mass index (BMI) was 26.8 ± 4.3 kg/m^2^ with a range of 17.4 to 38.6 kg/m^2^. As a whole, the subjects averaged 61,691 ± 25,274 steps over the seven-day period ranging from a minimum of 12,298 steps to a maximum of 148,180 steps. The daily step totals translated into an average of 8813 ± 3611 steps per day with a range of as few as 1757 steps to as many as 21,169 steps. Women averaged 8944 ± 3899 steps/day and men averaged 9220 ± 2330 steps/day (*P* = 0.758). Examination of the day-to-day variation in activity of the entire cohort revealed a sinusoidal pattern of activity level with step counts tending to rise from Monday to Tuesday then fall from Tuesday to a low point on Thursday, “mid-week” slump, followed by a rise again to a peak on Saturday with a fall again beginning from Saturday to Sunday. This day-to-day variability, while not statistically significant (*P* = 0.313), reflects the characteristic pattern for the group as a whole (Figure 2). 

 Pearson correlations revealed a significant positive correlation between gait speed and the mean number of steps per day (*r* = 0.530, *P* < 0.01). BMI was significantly negatively correlated with the mean number of steps per day (*r* = −0.337, *P* = 0.013). Age was not significantly correlated with average daily step count (*r* = 0.126, *P* = 0.364). To examine how age, gender, and BMI might explain variance in average daily step count when considered together, a backwards elimination regression analysis was conducted. Only BMI exhibited explanatory power in the regression model (*β* = −0.337, adjusted *r* ^2^ = 0.097, *P* = 0.013). 

 Evaluation of the activity levels of the group as a whole revealed an even distribution of the different activity groupings specified by Tudor-Locke and Bassett [13] (Table 1). When considered according to activity group, the “sedentary—low-activity” group was younger than the “somewhat active” group (*P* = 0.05) (Table 1), and had a higher BMI (*P* = 0.09) and walked significantly slower (*P* = 0.044) than the “active—highly active” group (Table 1). The “moderately active” group trended toward slower walking speeds compared to the “active—highly active group”, however this difference was not statistically significant (*P* = 0.059).

## 4. Discussion 

The present work describes activity levels, in terms of average daily step count for a specific subset of adults, that is, older adults between the ages of 45 and 70 who are without any evidence of clinical osteoarthritis in the hips and knees. To the best of our knowledge, activity levels for this specific subset of adults are not available in the existing literature. Our rationale for selecting this specific group was to provide a realistic benchmark for activity levels in patients after joint replacement. In the population of the present work, the average number of steps taken per day was close to 9000, which is approximately 4000 steps per day greater than has been previously reported in individuals after total joint arthroplasty [4–7] and approaches the recommended 10,000 steps suggested as a goal for physical fitness [8]. These data suggest that patients who undergo arthroplasty do not typically attain the same level of physical activity as age-matched adults without joint disease. The present findings differ from previous questionnaire-based reports that did not find such differences in physical activity after total hip arthroplasty (THA) patients when compared to age and sex-matched healthy adults [14, 15]. Two potential reasons for this difference are the method of data collection (i.e., accelerometer-based step counts versus questionnaire) and the younger age of the population studied in the present work. Franklin et al. reported that older age and high BMI increase the likelihood of poor functional gains after total knee arthroplasty [16]. In the present work, involving healthy adults between 45 and 68 years of age, those with a higher BMI tended to be less active; however, age did not explain variance in average daily step count. Our results are consistent with existing literature that suggests that BMI is inversely correlated with physical activity in patients after knee and hip replacement [17, 18].

 Accelerometer-based activity monitors have been used for the quantification of activity levels (based on step count) in healthy, osteoarthritis, and TJR populations [3, 18–23]. The high cost of these monitors is one of the main disadvantages when compared to low-cost waist-mounted pedometers; however, the availability of multiple locomotion parameters and their accuracy and the detailed information provided have to be considered in the overall cost-benefit comparison [19]. In this investigation the results of the AMP monitor validation showed a high accuracy (ICC 0.98) of step counts over a normal range of walking speeds (0.7–1.4 m/s). The lower-accuracy, but still acceptable, speed measurements (ICC 0.76) could be attributed to a not fully natural treadmill walking; from which monitor acceleration discrepancies may have been generated, affecting speed estimations. The limitations of treadmill walking are recognized; however they allow the analysis of locomotion parameters in a controlled manner [19].

 One limitation in this study is the fact that subjects knew their activity was being monitored and this potentially may have caused an artificial increase in activity level. In an effort to address this issue, subjects were required to wear the activity monitor for a period of seven consecutive days as opposed to one or two days immediately following the gait test, in the belief that a longer monitoring period might negate this effect. A second potential limitation of this work is the skewed distribution of males and females within the study population. When analyzed separately, the average daily step counts for men and women were not significantly different and thus the data from both genders were pooled. Females were not preferentially recruited over males. Female gender is a risk factor for developing knee osteoarthritis and thus one could argue the data from the current work might be more applicable to knee than hip arthroplasty.

 It is projected that patients under the age of sixty-five will account for more than 50% of all THA and total knee arthroplasty (TKA) patients by the years 2011 and 2016, respectively [10]. Specifically, the fastest growing age group for TKA is individuals aged 45–54, with the demand projected to increase 17-fold by 2030 [10]. In addition, candidates for joint replacements are reporting increased participation in regular physical activity as compared with those in decades past [24]. Research suggests patients' preoperative expectations for participation in leisure activities and recreational sports do not match their postoperative capabilities at 1 and 5 years after total knee replacement surgery [25]. While patients need to be encouraged to have realistic expectations for surgical outcomes, it is imperative to understand the activity level of adults in this age group who have pain-free, healthy knee, and hip joints, in order to provide a benchmark when designing rehabilitation programs to promote postoperative functioning. Ultimately, to reach the goal of returning such patients to a functional state comparable to their peers, as well as designing implants which can sustain such levels of physical functioning, a clearer understanding of activity levels in both the elderly and in patients who are progressively younger at the time of surgical intervention is required.

## Figures and Tables

**Table 1:** Comparison of demographic data for subjects when separated by activity classification.

	“Sedentary—low activity group” (<7500 steps per day)	“Somewhat active group” (7500–9999 steps per day)	“Active—highly active group” (>10,000 steps per day)	ANOVA
*N* (no. of subjects)	18	16	20	
Age (years)	51.1 ± 4.4*	57.6 ± 5.8*	54.1 ± 6.4	0.007
Body mass index (kg/m^2^)	29.3 ± 4.9*	26.0 ± 3.1	25.3 ± 3.8*	0.008
Gait speed (m/s)	0.88 ± 0.21*	0.88 ± 0.20	1.04 ± 0.18*	0.021

*Significant differences between groups (with Bonferroni *post hoc* test, *P* < 0.05).
